# Association of C5aR1genetic polymorphisms with coronary artery disease in a Han population in Xinjiang, China

**DOI:** 10.1186/s13000-015-0261-9

**Published:** 2015-04-18

**Authors:** Ying-Ying Zheng, Xiang Xie, Yi-Tong Ma, Yi-Ning Yang, Zhen-Yan Fu, Xiao-Mei Li, Shuo Pan, Dilare Adi, Bang-Dang Chen, Fen Liu

**Affiliations:** Department of Cardiology, First Affiliated Hospital of Xinjiang Medical University, Urumqi, 830054 P.R., China; Xinjiang Key Laboratory of Cardiovascular Disease Research, Urumqi, 830054 P.R., China

**Keywords:** C5aR1 gene, Single nucleotide polymorphism, Case–control study, Coronary artery disease

## Abstract

**Background:**

Complement 5a receptor (C5aR) was demonstrated a receptor of complement 5a (C5a) which is involved in many inflammatory diseases. The functional responses attributed to C5a results from its interaction with its receptors C5aR, which stimulates food intake, plays a role in increasing the inflammatory response in adipose tissue as well as the cardiovascular and neural systems. However, There are unknown associations between the SNPs of C5aR1 gene and coronary artery disease (CAD).

**Methods:**

We examined the role of the tagging single nucleotide polymorphisms (SNPs) of C5aR1 gene for CAD using a case–control design, and determined the prevalence of C5aR1 genotypes in 505 CAD patients and 469 age and sex-matched healthy control subjects of Han population.

**Results:**

The rs10853784 was found to be associated with CAD in dominant model (CC *vs* TT + CT, *P* = 0.004). The difference remained statistically significant after multivariate adjustment (*OR* = 1.430, 95% *CI*: 1.087 ~ 1.882, *P* = 0.011). There was no significant difference in genotype distributions of rs4577202 and rs7250152 between CAD patients and control subjects. The frequency of the haplotype (A-T-C) was significantly higher in the CAD patients than in the controls (*P* = 0.035), and the haplotype (A-C-T) was significantly lower in the CAD patients than in the control subjects in Chinese Han population (*P* = 0.002).

**Conclusion:**

The results of this study indicate that rs10853784 of C5aR1 gene are associated with CAD in Han population of China, and A-C-T haplotypes may be protective genetic marker and the A-T-C may be risk genetic marker for CAD in Chinese Han population.

**Virtual slides:**

The virtual slide(s) for this article can be found here: http://www.diagnosticpathology.diagnomx.eu/vs/2054871241495194.

## Background

The role of the immune system and inflammatory pathways in the development of atherosclerotic disease is well established [1]. Recent evidences claimed that inflammation might represent the pathophysiological link between obesity and metabolic syndrome (MetS) [2]. Since adipose tissue is now considered an endocrine organ, affecting metabolism and vascular function. The understanding of these pathways is crucial from a pathophysiological point of coronary artery disease (CAD) risk stratification and to the identification of possible therapeutic targets. Disorders of lipoprotein metabolism such as elevated levels of triglyceride (TG), Lipoprotein (a) [Lp(a)] as well as increased fasting blood glucose are considered to be important risk factors in the pathogenesis of atherosclerosis and CAD [3-6]. CAD is a chronic inflammatory disease. There is evidence on the role of inflammation in all stages of the atherosclerotic disease process [7,8]. Genetic studies have revealed many causal or susceptible inflammatory loci associated with CAD [9,10].

C5a is a multifunctional protein, which promotes the recruitment and activation of neutrophils and monocytes [3]. Pathological conditions such as sepsis and various immunoinflammatory disorders are accompanied by increases in circulating C5a [11-13]. C5a interacts primarily with its two receptors, the classic proinflammatory C5a receptor (C5aR; CD88) and relative newly identified C5a receptore like 2 (C5L2). C5aR is a member of the rhodopsin family of G protein-coupled receptors (GPCR) which is expressed at varying levels in different immune and non-immune cells [14-17]. A recent research demonstrated that the C5aR Knockout(KO) mice have decreased fat mass, glucose clearance and insulin tolerance [18]. The C5a-C5aR pathway has been targeted for pharmacological therapy for treatment of sepsis, cardiovascular diseases, and autoimmune disorders [19,20]. C5L2, which resembles C5aR (58% homology) [12] was demonstrated to be a functional receptor of acylation-stimulating protein (ASP), increased transport of glucose and esterification of fatty acids, leading to a net accumulation of TG stores influence the body’s susceptibility to CAD [21-23].

In previous studies [24-26], we identified C5L2 gene which is associated with CAD and T2DM in Chinese Han and Uygur population. The activity of C5L2 may influence the individual’s susceptibility to CAD. Accordingly, we screened for tagging SNPs of the C5aR1 gene and assessed the association between the genotypes of this gene and CAD in Chinese Han population.

## Methods

### Ethical approval of the study protocol

Written informed consent was obtained from all participants. An approval of ethics committee by the Ethics Committee of the First Affiliated Hospital of Xinjiang Medical University (Xinjiang, China) was obtained. It was conducted in accordance with the guidelines of the Helsinki Declaration.

### Subjects

Participants diagnosed with CAD were recruited at the First Teaching Hospital of Xinjiang Medical University from 2007 to 2010. The detailed diagnostic and selection criteria have been previously described [27,28]. Briefly, CAD was defined as the presence of at least one significant coronary artery stenosis of ≥50% luminal diameter on coronary angiography. Patients were excluded if they had congenital hypercoagulable status with proven disease-limiting life expectancy or had abused cocaine. The healthy participants were selected from the cardiovascular risk survey (CRS). The CRS has been described previously [29]. This study consists of 14,618 subjects and is a multiple-ethnic, community-based, cross-sectional study designed to investigate the prevalence, incidence, and risk factors for cardiovascular diseases in the Han, Uygur, and Kazakh population in Xinjiang (west China) between June 2007 and March 2010. These control individuals did not have a history of CAD, electrocardiographic signs of CAD, regional wall motion abnormalities, and relevant valvular abnormalities in echocardiograms [30].

In this study, 505 patients with CAD and 469 healthy control participants were enrolled. All study participants were Han Chinese ethnicity and were from the same geographic area in Xinjiang. Demographic data and subject characteristics, including hypertension, diabetes mellitus, smoking, alcohol consumption, and serum cholesterol, were collected for all study participants. Diabetes, hypertension, hyperlipidemia, smoking, and alcohol consumption were defined as described previously [27-29].

### Biochemical analysis

Serum concentrations of total cholesterol (TC), TG, glucose, high-density lipoprotein cholesterol (HDL-C), low-density lipoprotein cholesterol (LDL-C), apolipoprotein B (Apo B), LP (a) were measured using standard methods in the Central Laboratory of First Affiliated Hospital of Xinjiang Medical University as described previously [27-29].

### Sample DNA extraction

Blood samples were collected from all participants using a standard venipuncture technique and EDTA-containing tubes. DNA was extracted from the peripheral blood leukocytes using a whole blood genome extraction kit (Beijing Bioteke Corporation, Beijing, China. (http://bioteke.technew.cn) as previously described [27,29].

### Genotyping of C5aR1 gene

The genotyping for the C5aR1 gene was performed by using ABI 7900 Real Time PCR System (Applied Biosystems). Amplification was performed in 6ul of volume, including 1ul of DNA, 3ul of Master Mix, 0.05ul of Probe(40X), MiliQ add to 6ul. Real time PCR was performed according to the protocol as follows: After an initial holding step of Pre-heated 95°C for 10 min, samples were cycled 20 times at 95°C for 10s, at 65°C-55°C for 15 s (Each loop drops by 0.5°C), at 60°C for15s. Then the samples were cycled 25 times at 95°C for 10s, at 60°C for 30s, pre/post read (Starting from 60°C at 0.06°C/s was slowly warmed to 61°C, and collecting the fluorescence value). Cooling (cool at 40°C to maintain).

### Statistical analyses

Statistical analysis was performed using the SPSS version 17.0 software (SPSS, Chicago, IL, USA), Data are expressed as the mean ± standard deviation (SD). The significance of differences was evaluated using the t-test for continuous variables and the χ^2^ test for non-continuous variables. The differences between CAD patients and control subjects were assessed by independent-sample t-test. Categorical variables were analyzed as frequencies. Allele and genotype frequencies among CAD cases and controls were compared with values predicted by using the Chi-square test. Hardy-Weinberg equilibrium was assessed by chi-square analysis. Multivariate analysis was performed using a logistic regression analysis for independent variables that were related to the presence or absence of CAD. A value of *P* < 0.05 was considered significant.

## Results

### Characteristics of participants

Table 1 shows demographic and clinical characteristics of 974 study subjects. CAD patients (n = 505) and healthy control subjects (n = 469), the following variables were significantly different between the two groups: diabetes and drinking; the serum concentration of glucose, apo B and LP(a) (all *P* < 0.05). There was no significant difference in the following variables between CAD patients and control subjects: hypertation; smoking, the body mass index (BMI); serum concentration of TG, HDL, and LDL, age, and sex (all *P* > 0.05).

**Table 1:** **Characteristics of the participants**

**Characteristics**	**CAD (n = 505)**	**Control (n = 469)**	***χ2*** **or** ***t***	***P*** **value**
Age, mean (SD)	61.91 (9.94)	57.31 (10.27)	1.631	0.202
Sex, female (%)	169 (33.46)	159 (33.90)	0.019	0.47
Hypertension, n (%)	239 (47.33)	208 (44.35)	0.868	0.368
Diabetes, n (%)	105 (20.79)	39 (8.31)	30.045	<0.001
Smoking, n (%)	47 (9.30)	47 (10.02)	0.142	0.745
Drinking, n (%)	27 (5.34)	42 (8.96)	4.811	0.033
BMI, mean (SD)	25.50 (3.277)	25.90 (3.40)	0.896	0.344
Apo B, mean (SD)	1.02 (2.87)	0.84 (0.23)	4.033	0.045
Lipoprotein(a), mean (SD)	203.53 (190.51)	170.42 (149.69)	17.673	<0.001
TG, mean (SD)	2.87 (13.71)	1.94 (1.54)	3.46	0.06
TC, mean (SD)	4.27 (1.15)	4.35 (1.03)	2.907	0.089
HDL-C, mean (SD)	1.53 (7.77)	1.13 (0.35)	3.716	0.054
LDL-C, mean (SD)	3.69 (13.09)	3.20 (13.64)	0.999	0.318
Glucose, mean (SD)	6.25 (2.60)	5.56 (1.77)	35.393	<0.001

Note: Continuous variables are expressed as mean ± SD. Categorical variables are expressed as percentages. BMI, body mass index; TG, triglyceride; TC, total cholesterol; HDL-C, high density lipoprotein cholesterol; LDL-C, low density lipoprotein cholesterol; The P value of the continuous variables was calculated by the Independent t-test. The P value of the categorical variables was calculated by Fisher’s exact test.

### Distribution of the SNPs for C5aR1 gene in CAD patients and controls

Table 2 shows distribution of genotypes and alleles of SNPs for C5aR1 gene. The genotypes distributions for each of the SNPs were in good agreement with the predicted Hardy-Weinberg equilibrium values (data not shown). There was no significant difference between CAD and control subjects for rs4577201 (SNP1) and rs7250152 (SNP2) (all *P* > 0.05). Distribution of rs10853784 (SNP3) genotypes, dominant model (CC vs TT + CT), additive model (CT vs CC + TT) and allele frequency showed significant difference between CAD and control subjects (*P* = 0.016, *P* = 0.004, *P* = 0.021 and *P* = 0.004, respectively). There was no significant difference between CAD and control subjects for recessive model (TT *vs* CC + CT, *P* = 0.191).

**Table 2:** **Genotype and allele distributions in patients with CAD and control participants**

**rs4577202(SNP1)**	**rs7250152(SNP2)**	**rs10853784(SNP3)**									
**Variant**	**CAD n(freq)**	**Control n(freq)**	***P*** **value**	**Variant**	**CAD n(freq)**	**Control n(freq)**	***P*** **value**	**Variant**	**CAD n(freq)**	**Control n(freq)**	***P*** **value**
Genotype				Genotype				Genotype			
AA	325(0.644)	309(0.658)	0.814	TT	166(0.328)	132(0.282)	0.277	TT	20(0.040)	27(0.058)	0.016
AG	156(0.309)	141(0.301)		CT	237(0.470)	234(0.499)		CT	151(0.299)	173(0.369)	
GG	24(0.047)	19(0.041)		CC	102(0.202)	103(0.219)		CC	334(0.661)	269(0.574)	
Dominant model				Dominant model				Dominant model			
AA	325(0.644)	309(0.658)	0.617	TT	166(0.328)	132(0.282)	0.110	CC	334(0.661)	269(0.574)	0.004
AG + GG	180(0.356)	160(0.342)		CT + CC	339(0.672)	337(0.719)		CT + TT	170(0.339)	200(0.426)	
Recessive model				Recessive model				Recessive model			
GG	24(0.047)	19(0.041)	0.575	CC	102(0.202)	103(0.219)	0.500	TT	20(0.040)	27(0.058)	0.191
AA + AG	481(0.953)	454(0.959)		CT + TT	403(0.798)	366(0.781)		CT + CC	485(0.960)	442(0.942)	
Additive model				Additive model				Additive model			0.021
AG	156(0.309)	141(0.301)	0.779	CT	237(0.470)	234(0.499)	0.335	CT	151(0.299)	173(0.369)	
AA + GG	349(0.691)	328(0.699)		CC + TT	268(0.530)	235(0.501)		CC + TT	354(0.701)	296(0.631)	
Allele				Allele				Allele			
A	806(0.799)	759(0.809)	0.536	T	569(0.563)	498(0.532)	0.150	T	191(0.193)	227(0.241)	0.004
G	204(0.201)	179(0.191)		C	441(0.437)	440(0.468)		C	819(0.807)	711(0.759)	

Note: Freq, Frequency; CAD, Coronary artery disease; N, number of participants; SNP, single-nucleotide polymorphism.

Table 3 shows multivariable logistic regression analysis combining genotypes with following variables: plasma concentration of lipoprotein(a), incidence of diabetes, drinking and smoking which were the major confounding factors for CAD. After multivariate adjustment, rs10853784 remain significantly associated with CAD in dominant model (CC *vs* CT + TT) (*OR* = 1.430, 95% confidence interval [*CI*]:1.087 ~ 1.882, *P* = 0.011).

**Table 3:** **Multiple logistic regression analysis for CAD patients and control subjects**

	***B***	***S.E.***	**χ** ^***2***^	***P***	***OR***	***95% CI***
Dominant model (CC vs CT + TT) (SNP3)	.358	.140	6.537	.011	1.430	1.087 ~ 1.882
Diabetes	1.059	.210	25.442	<0.001	2.885	1.911 ~ 4.354
Smoking	.617	.333	3.442	.064	1.854	0.966 ~ 3.559
Drinking	−1.053	.388	7.356	.007	.349	0.163 ~ 0.747
Lipoprotein(a)	.001	.000	6.068	.001	.229	1.000 ~ 1.002
Constant	−1.473	.438	11.328	.001	.248	

### Haplotypes of the C5aR1 Gene Associated with CAD

In the haplotype-based case–control analysis, haplotypes were established through the use of different combinations of the SNPs (Table 4). The overall distribution of the haplotypes established by SNP1-SNP2-SNP3 was significantly different between the CAD patients and the control subjects. The frequency of the A-C-T haplotype established by SNP1-SNP2-SNP3 in these two groups was also significantly lower for the CAD patients as compared to the control subjects (*OR* = 0.714, 95% *CI* = 0.577-0.883, *P* = 0.002). Moreover, the frequency of the A-T-C haplotype established by SNP1-SNP2-SNP3 in these two groups was also significantly higher for the CAD patients compared to the control subjects (*OR* = 1.213, 95% CI = 1.014-1.452, *P* = 0.035).

**Table 4:** **Haplotype analysis in patients with CAD and control subjects**

**Haplotype**	**SNP1**	**SNP2**	**SNP2**	**Case(freq)**	**Control(freq)**	**χ2**	**P value**	**Odds ratio [95% CI]**
H1	A	C	C	243.50(0.229)	221.08(0.218)	0.671	0.413	1.090 [0.886 ~ 1.342]
H2	A	C	T	192.89(0.182)	244.07(0.241)	9.701	0.002	0.714 [0.577 ~ 0.883]
H3	A	T	C	412.31(0.388)	354.84(0.351)	4.441	0.035	1.213 [1.014 ~ 1.452]
H4	G	T	C	183.38(0.173)	181.83(0.180)	0.05	0.823	0.974 [0.777 ~ 1.222]

## Discussion

Factors such as diabetes, hypertension, inflammation and hyperlipidemia have been reported to influence the pathogenesis of CAD. Like hyperlipemia and diabetes, CAD is thought to be a multifactorial disease. Hence, much attention has been focused on the association of gene polymorphisms with CAD.

The C5aR and C5L2, was identified in 1991 [31,32] and 2000 [33], respectively. Both genes are localized to the same region of chromosome 19, q13.33 and encoded in a two exon structure, with the 5′-untranslated region and initiating codon in the first exon, and the remainder of the coding sequence and the 3′-untranslatedregion in the second [34]. This is typical of the members of the chemoattractant receptor family. In the promoter region of C5aR, an SNP at position-245 (T/C) has been discovered [35] and the coding region. C5aR has two non-synonymous SNPs at 4G/A (Asp/Asn at amino-acid position 2) and 859G/T (Asn/Lys at position 278) and two synonymous SNPs: 72 T/C and 727G/A [36]. To date, no SNPs have been identified in the coding region of the C5aR1 gene association with human disease. C5aR, which binds C5a, is involved in many inflammatory diseases [37]. One previous study showed that C5a stimulates food intake [38], the C5aR KO mice demonstrated decreased body weight and fat storage [22]. Studies earlier showed the higher levels of C5a was associated with late lumen loss of drug-eluting stents [39,40]. Early atherosclerotic lesions express C5aR, which may indicate that inflammatory cells are initially recruited into the damaged vessels through C5aR [41]. Other research reported that C5a concentration was more elevated in hypertensive individuals than in healthy people. C5aR signaling pathway on blood monocytes/macrophages plays a pathological role in angiotensin II (Ang II)-induced cardiac inflammation and remodeling. Complement proteins such as complement C3 [42,43], C5L2 [44,45] and C3aR [46] have all been implicated in lipid and glucose metabolism. C5L2 is similar to C5aR in structure, C5L2 was associated with hyperlipidemia and diabetes, and the C5L2 genetic variant was associated with CAD and this association is not modified by the concentration of TG and glucose.

In our study, we found that polymorphisms of C5aR1 were associated with CAD in a Han population. There was significant difference in genotype distribution of rs10853784 between CAD patients and control subjects. For the rs10853784 genotypes, dominant model (CC vs TT + CT), additive model (CT vs CC + TT) and allele frequency showed significant difference between CAD and control subjects. After multivariate adjustment of confounding factors the significant difference was retained.

For genes with multiple susceptibilities, an analysis based on haplotypes has advantages over an analysis based on individual SNPs, particularly when the linkage disequilibria between the SNPs is weak [47]. Our study is the first haplotype-based case–control study to investigate the association between the human C5aR1 gene and CAD in the Chinese Han population. In our study, we succeeded in identifying two haplotypes (A-C-T and A-T-C) of SNP1-SNP2-SNP3 in Chinese Han population. Based on the haplotype and logistic regression analyses, we believe that the haplotype (A-C-T) is a protective factor for CAD (*OR* = 0.714, *P* = 0.002), and A-T-C is an risk factor for CAD (*OR* = 1.213, *P* = 0.035) in Chinese Han population.

## Conclusion

In conclusion, this is the first time that correlations between the human C5aR1 gene and CAD have been examined in the Chinese population. The present data indicate that C5aR1gene is associated with CAD in Han population of China. This result may broaden the knowledge of genetic variants and disease-association studies.

### Study limitation

The present study was limited by the relatively small sample size. This may have led to weak statistical significance and wide CIs when estimating ORs.
